# A Simplified Daily Fit Model to Reduce Costs and Nutrient Intake in Growing-Finishing Pigs

**DOI:** 10.3390/ani14202922

**Published:** 2024-10-11

**Authors:** Yann M. Ferreira, Rayna S. V. Amaral, Blandina G. V. Silva, Leila C. S. Moura, Diana A. Oliveira, Tadeu E. da Silva, Luciano Hauschild, Ines Andretta, Luan S. Santos

**Affiliations:** 1 Graduate Program in Animal Science, Universidade Federal do Rio de Janeiro, Seropédica 23890-000, Rio de Janeiro, Brazil; yannmalini@yahoo.com (Y.M.F.); rayna.amaral@hotmail.com (R.S.V.A.); blandinagvs@gmail.com (B.G.V.S.); mouraleila@ufrrj.br (L.C.S.M.); assis.diana@outlook.com (D.A.O.); 2 Department of Animal & Veterinary Sciences, University of Vermont-Burlington, Burlington, VT 05405, USA; tdasilva@uvm.edu; 3 Faculdade De Ciências Agrárias e Veterinárias, Unesp Universidade Estadual Paulista, Jaboticabal 14884-900, São Paulo, Brazil; luciano.hauschild@unesp.br; 4 Faculdade de Agronomia, Universidade Federal do Rio Grande do Sul, Porto Alegre 91540-000, Rio Grande do Sul, Brazil; ines.andretta@ufrgs.br; 5 Faculdade de Medicina Veterinária e Zootecnia, Universidade Federal de Mato Grosso do Sul, Campo Grande 79070-900, Mato Grosso do Sul, Brazil

**Keywords:** feed cost reduction, nutrient management, precision feeding, sustainable pig farming, swine nutrition

## Abstract

**Simple Summary:**

The study focuses on improving how we feed pigs to reduce costs and be more environmentally friendly. Traditionally, pigs are fed in phases with their diet changing at set growth stages. This often leads to overfeeding and waste. A new method called the daily fit model (DFM), which adjusts the diet daily based on the pigs’ needs, was explored. By comparing this new approach with the traditional method, it was found that the DFM significantly reduces the intake of key nutrients like protein and phosphorus without affecting the pigs’ growth. This means lower costs for farmers and less environmental impact from pig farming. The simplified approach makes it easier for farmers to adopt precision feeding without advanced technology or extensive training. This method can help farmers save money, reduce waste, and promote sustainable farming practices.

**Abstract:**

Precision feeding is an excellent alternative to conventional phase feeding systems for growing-finishing pigs, especially with increasing feeding costs and environmental sustainability concerns. However, precision feeding strategies sometimes require advanced technologies such as electronic devices and the modernization of pig facilities. In addition to hardware implementation, precision feeding is frequently related to mathematical difficulties due to a lack of professionals trained in decision making. Therefore, this study compares a conventional phase feeding model (CON) and a daily fit model (DFM) with a simplified approach to the conscious use of nutrients for pig production. A simulation study was conducted using growth curves of barrow pigs, following three nutritional recommendations for conventional phase feeding. Once the nutrient requirements for CON were determined, these diets were used for the DFM by anticipating a proportional percentage of the next phase diet in the current diet. This simple adjustment does not impair the growth performance of pigs. However, in this study, the DFM showed promise during the growing-finishing phases to reduce pigs’ costs and nutrient intakes, such as crude protein, lysine, and digestible phosphorus, up to 5.58, 7.11 and 9.13%, respectively. In conclusion, the DFM can effectively reduce costs, minimize environmental impact, and promote sustainable practices. Also, this industry-wide adoption of this simplified precision feeding strategy could play a vital role in swine farmers’ challenges, fostering broader environmental benefits and improved resource efficiency.

## 1. Introduction

Precision feeding (PF) is a strategic approach to curtailing feed expenditures, setting itself apart from the conventional phase feeding system. This approach aims to provide pigs with the correct quantity and composition of feed at the right moment [1]. Conversely, phase feeding entails altering the nutrient content of pig diets during various stages or phases of their growth to better align with their evolving daily nutritional needs [2]. This strategy causes a nutrient oversupply, especially in pigs with lower nutrient requirements [3]. The economic advantages of precision feeding are substantial, particularly within growing-finishing pig facilities [1,4,5,6,7]. Variability among individual pigs due to factors such as age [8], sex, and genetics leads to significant differences in their nutritional requirements [9,10].

Despite advanced research on the benefits of PF for pigs, some challenges still need to be addressed. The high cost of adoption, technology-related difficulties, lack of professional support, and lack of supporting policies are farmers’ main concerns regarding adopting precision technologies [11]. Other more complex models rely on real-time data collection and analysis, often necessitating electronic feeding systems and individualized monitoring. These advanced technologies make it difficult for small producers to adopt such practices, leaving a gap in practical, accessible solutions for the broader industry. Also, workers must be trained to use these tools and evaluate the data collected [12].

This simple model can help farmers adopt new technologies because it is less complex than other precision models. The model uses a group-based feeding strategy that anticipates dietary adjustments, avoiding the need for complex technologies and individual animal monitoring. This model innovates by being cost-effective and more accessible to implement. It can be used in a simple Excel spreadsheet. With the financial and logistics challenges of precision feeding approaches [13], strategic decisions to adopt these technologies have to be made.

In growing-finishing pig facilities, precision feeding reduces the surplus nutrients in pig diets [6]. Reduced phosphorus (P) intake in the feed decreases P excretion [14]. Furthermore, studies have shown that a reduction in dietary protein intake leads to a corresponding decrease in nitrogen (N) excretion [15,16].

Given the merits of both feeding strategies, this project aims to compare the economic advantages and nutrient reduction achieved by a conventional five-phase feeding model (CON) with those of a daily adjustment model (DFM). While the CON employs distinct diets in each feeding phase, the DFM adapts feed provision to growing-finishing pigs simply by gradually anticipating their next diet.

## 2. Materials and Methods

### 2.1. Data Collection and Scenario Definition

In this study, data from 11 feeding curves of pigs from four distinct sex categories, with varying initial weights, daily feed intakes (ADFIs), and average daily gain (ADG), were collected. Specifically, animal growth data were obtained from barrows with an initial body weight of 20.61 ± 0.85 kg, reaching a final body weight of 138.94 ± 0.90 kg over a 120-day growing-finishing period. The feed intake and growth curves were obtained from a comprehensive database containing records of over 1,000,000 animals. Three operational pig farms provided commercial data. Pigs were kept in commercial conditions (ad libitum access to water and feed, group housing, ambient room temperature of 20–24 °C) for 120–150 days. All collected data were tabulated for subsequent analysis and modeling purposes. Statistical analysis was not required, since the study is based on predefined models and deterministic inputs rather than empirical data collection.

Three distinct scenarios were divided, adhering to the barrow requirements outlined in the Brazilian tables for poultry and swine [17], the National Research Council [18], and the commercial lineage AGPIC [19]. These scenarios assessed and compared two feeding models: the conventional 5-phase feeding model (CON) and the daily fit model (DFM).

For each scenario, ADFI and ADG data from the barrows were employed to evaluate the two feeding systems: the 5-phase system, which entails supplying the same diet to all pigs within the group during each proposed phase, and the daily feeding system, which adjusts the diet based on the nutritional requirements of pigs as they age. The daily feeding system anticipates the subsequent diet through daily DFM adjustments. In the simulations, five feed phases were considered, each with varying durations in days, which were determined by the weight range of the animals (please check Table 1 for details).

### 2.2. Model Description

Two models were employed to facilitate a comparison of the feed systems. The first model (1) calculates feed costs in the CON, taking into account the phase duration (D), feed price (F) within each phase, and feed intake during the respective phase (i). The total cost is derived by summing the costs of all phases, depending on the number of feeding phases employed (P).

Ccf = ∑i = P (D × F × I)(1)

The second model (2) is designed to optimize daily feed intake and nutrient provision rather than adhering to fixed feeding phases as in the CON. The DFM continuously adjusts the diet to match the pigs’ precise nutritional requirements as they grow, aiming to reduce feed costs and nutrient waste while enhancing overall production efficiency. It considers the feed cost (Ctc) and daily feed intake (DFI). Calculating the total cost value (Ctc) requires knowledge of the amount of feed intake (AFI) and the corresponding feed prices (FP) (3).

This adjustment is based on the pigs’ current growth stage, nutrient requirements, and the feed prices available at each point. The pigs’ daily feed intake is monitored and used to calculate the amount of feed needed for each day, which is then multiplied by the corresponding feed cost to determine the daily feed cost. The total cost is the sum of the daily costs over the entire production period. A summary of these models is presented in Table 2.

Cda = ∑i = P (Ctc × DFI)(2)

Ctc = (AFI1 × FP1) + (AFI2 × FP2)(3)

These models can also be applied to predict nutrient reduction. Instead of price, the input is adjusted to reflect the quantity of nutrients in the diet. After model construction, results were compiled, organized, and tabulated in a Microsoft Excel^®^ (Version 2307) spreadsheet to create a comprehensive database and facilitate the development of models for calculating excess nutrient reduction in diets and feed costs. All simulations and model calculations were performed using Microsoft Excel, and data visualization was performed using the matplotlib (version 3.9.0) library for the Python programming language. The automated spreadsheet was designed to efficiently calculate feed costs and nutrient reduction across different scenarios.

### 2.3. Formulation of Virtual Feeds

Diets were formulated employing the solver procedure available in Microsoft Excel. For the simulations, six diets were devised, which were guided by the nutrient requirements for barrows established in the Brazilian tables for poultry and swine [17], NRC [18], and AGPIC [19]. The sixth feed diluted the fifth feed with details on the diets provided in Supplementary Tables S1 and S2.

### 2.4. Simulation Study

Three models were employed to estimate the requirements of SID Lys: BT-2017 from the Brazilian Tables, the NRC-2012 model, and the AGPIC-2021 model. The NRC-2012 model underwent slight modifications to enhance comparability between the models, which were akin to the approach in [20]. The simulated Metabolizable Energy (ME) content was set at 3.4 Mcal.

BT-2017 Model

SID Lys requirement (g/day) = 0.036 × BW^0.75^ + Y × ADG (4)

where Y = 16.664 + 0.0736 × BW − 0.0003 × BW^2^

NRC-2012 Model

Lys losses (g/day) = DFI × 0.417 × 0.88 × 1.1(5)

Integument Lys losses (g/day) = 0.0045 × BW^0.75^ (6)

SIDLysM (g/day) = [((Equation (2) + Equation (3))/(0.75 + 0.002)) × (Maximum PD − 147.7)](7)

Lys retained in PD (g/day) & Non − ractopamine − induced = (PD × 7.10)/100(8)

SIDLysG (g/day) = {(Lys retained in PD)/([0.75 + 0.002 × (maximum PD − 147.7)])}/(1 + 0.0547 + 0.002215 × BW)(9)

Pd barrows (g/day) = (133) × (0.7078 + 0.013764 × BW − 0.00014211 × BW^2^ + 3.2698 × 10^−7^ × BW^3^) (10)

AGPIC-2021 Model

SID Lys for barrows if weight is <40 kg = 0.0000255654 × (weight, kg × 2.204622)^2^ − 0.0157978368 × (weight, kg × 2.204622) + 4.4555073859 (11)

SID Lys for barrows if weight is >40 kg = Equation (11) + (−0.0000000031 × (weight, kg + 0.0000013234 × weight, kg^3^ − 0.0002087068 × weight, kg^2^ + 0.0142221655 × (weight, kg − 0.3126825057] × Equation (11) (12)

In addition to SID Lys intake comparisons, weekly calculations were conducted for crude protein (CP) intake, assessing differences between the CON and DFM. The evaluation of SID Lys intake compared the percentage of SID Lys in the daily diet with the requirement for each scenario on the first day of each feeding phase. Moreover, Cumulative CP, Amino Acid (AAC), Total Nitrogen (N), and standardized total tract digestible Phosphorus (STTD P) were compared across the models. Finally, the disparity in feed costs ($) between the application of the CON and DFM was analyzed. The conversion from Brazilian real to US dollars was performed using an exchange rate of 5.05 real per dollar.

## 3. Results

The results of CON and DFM simulations analyzed as described above are shown for NRC-2012, BT-2017, and AGPIC-2021. The primary objective is to model and compare the two feeding systems’ nutrient intake and cost implications (CON and DFM) under controlled conditions.

### 3.1. SID Lys Requirements

**Figure 1:**
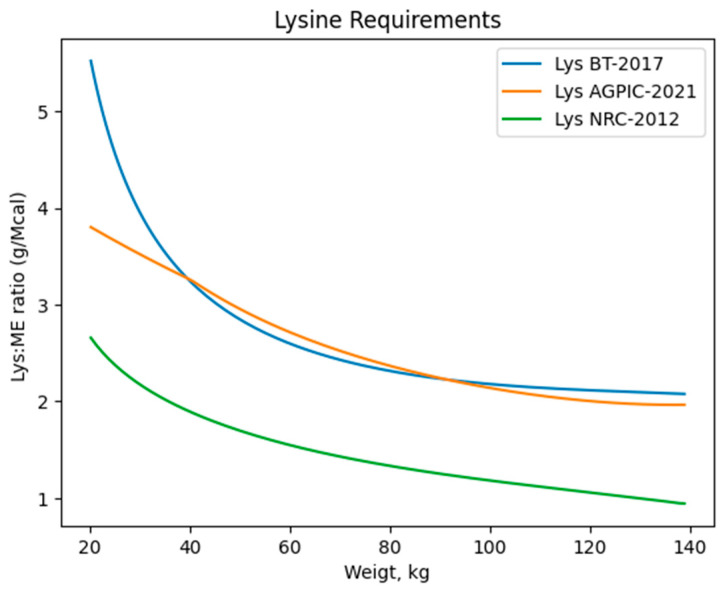
Lysine requirements for growing-finishing barrows following the Brazilian Tables (BT-2017), NRC (NRC-2012), and AGPIC (AGPIC-2021) requirements. The simulated Metabolizable Energy (ME) content was set at 3.4 Mcal.

The nutrient requirements from different recommendations between NRC-2012, BT-2017, and AGPIC-2021 showed essential variations in curves of SID Lys to calorie ratio of ME. These factorial methods provide valuable estimations of nutrient requirements for pigs reared in large groups and subjected to extended periods of uniform feed consumption throughout their production cycle (Figure 1).

### 3.2. Crude Protein and Amino Acid Intake

Across all scenarios, our simulations consistently showed reduced nutrient intake when employing the DFM compared to the CON. This reduction encompassed essential components such as protein, amino acids, and phosphorus, ultimately decreasing feed costs.

**Figure 2:**
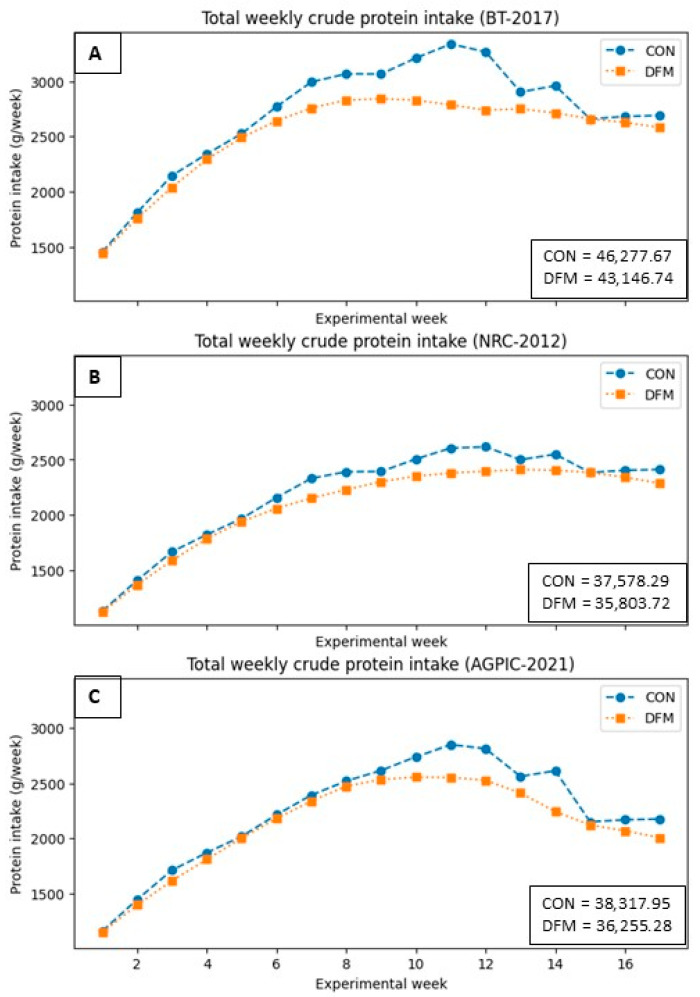
Total weekly crude protein intake in the proposed scenarios. (**A**): BT-2017; (**B**): NRC-2012; (**C**): AGPIC-2021. Abbreviations: CON = conventional phase feeding model; DFM = daily fit model.

**Figure 3:**
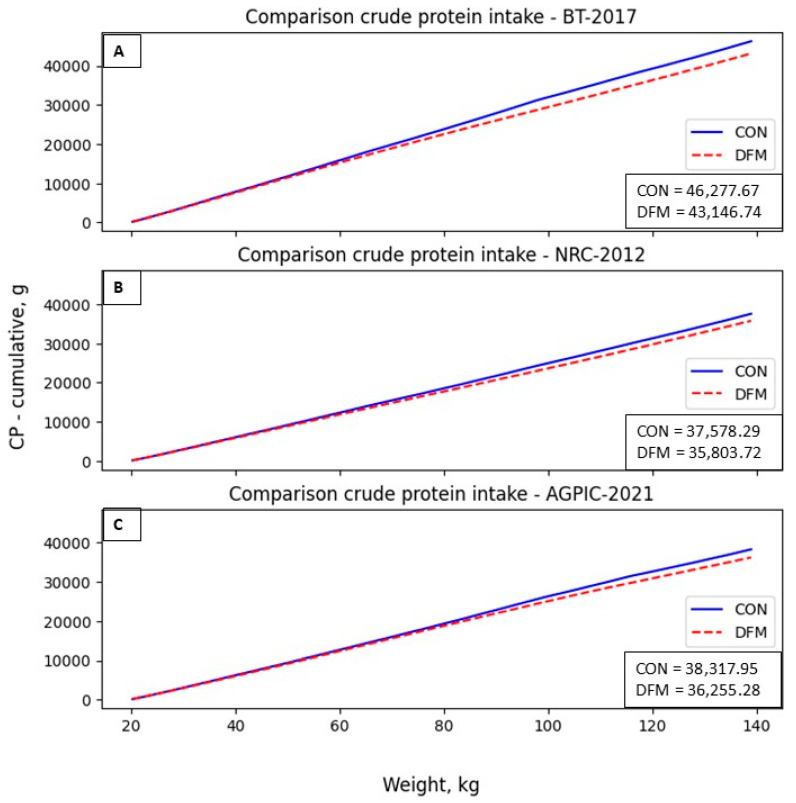
Cumulative crude protein intake (g) in the diet in the proposed scenarios. (**A**): BT-2017; (**B**): NRC-2012; (**C**): AGPIC-2021. Abbreviations: CON = conventional phase feeding model; DFM = daily fit model.

Figure 2 illustrates changes in weekly crude protein (CP) consumption between DFM and CON, showing a more significant reduction in the BT compared to NRC and AGPIC scenarios. The total CP intake accumulated also shows reductions (Figure 3) for the BT, NRC, and AGPIC of 6.77, 4.72, and 5.38% respectively. The period of these differences was also varied among the modeled scenarios. The reductions of CP in the BT and NRC scenarios started from the 6th week and coincided with the transition between phase 2 and phase 3. In the AGPIC scenario, the DFM initiated CP reductions after nine weeks (during the shift from phase 2 to phase 3). These reductions were most pronounced during the 11th week in BT and NRC, with differences of 552 g and 225 g, respectively, and during the 14th week in the AGPIC scenario, with the most substantial difference amounting to 369 g. These discrepancies corresponded with feed phases 4 and 5, where the DFM’s ability to align CP requirements with the pigs’ actual needs closely resulted in enhanced CP intake reduction.

**Figure 4:**
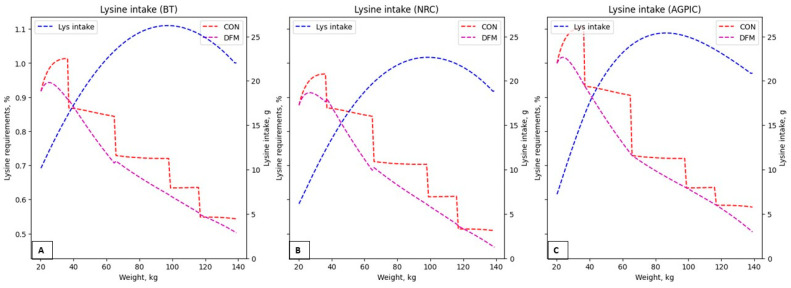
Lysine in the feed (%) in the proposed scenarios and lysine requirements following the Brazilian tables (BT-2017), NRC (NRC-2012), and AGPIC (AGPIC-2021) requirements and lysine intake (g). (**A**): BT-2017; (**B**): NRC-2012; (**C**): AGPIC-2021. Abbreviations: CON = conventional phase feeding model; DFM = daily fit model; Lys intake = lysine requirements.

**Figure 5:**
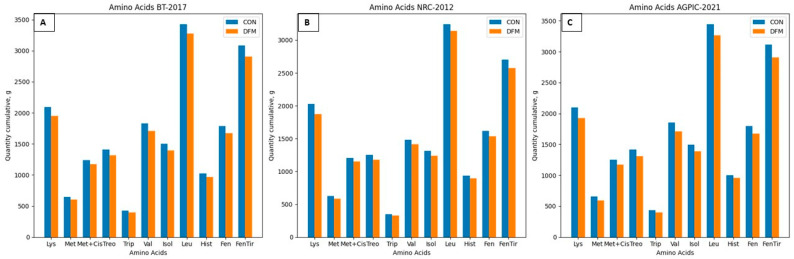
Cumulative amino acid intake (g) in the proposed scenarios. (**A**): BT-2017; (**B**): NRC-2012; (**C**): AGPIC-2021. Abbreviations: CON = conventional phase feeding model; DFM = daily fit model.

When comparing the diets’ lysine content among the scenarios (Figure 4), it becomes evident that applying the DFM consistently leads to lower lysine intake across all scenarios. Similar reductions are observed in the diet’s levels of other essential amino acids with a decrease in quantity evident when employing daily adjustment models (Figure 5).

### 3.3. Total Nitrogen and Phosphorus Intake

**Figure 6:**
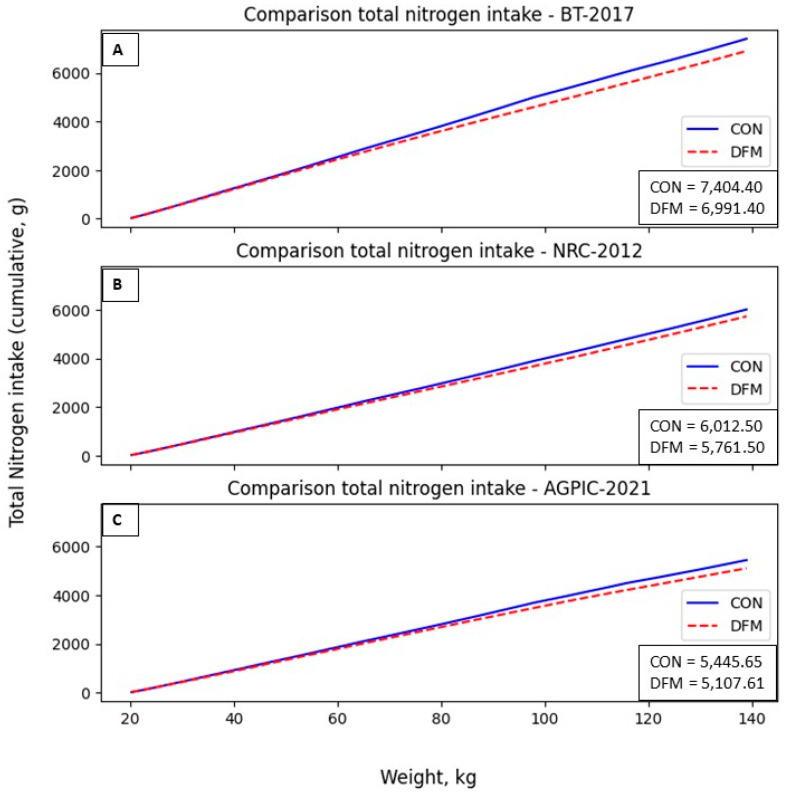
Total nitrogen intake (g) in the proposed scenarios. (**A**): BT-2017; (**B**): NRC-2012; (**C**): AGPIC-2021. Abbreviations: CON = conventional phase feeding model; DFM = daily fit model.

The CON showed that pigs consumed more CP in all scenarios. In contrast, the DFM reduced the CP in the diet and, consequently, the CP intake of pigs. In the BT, NRC, and AGPIC scenarios, the daily adjustment model reduced total accumulated dietary nitrogen by 6.77%, 4.72%, and 6.21%, respectively (Figure 6).

**Figure 7:**
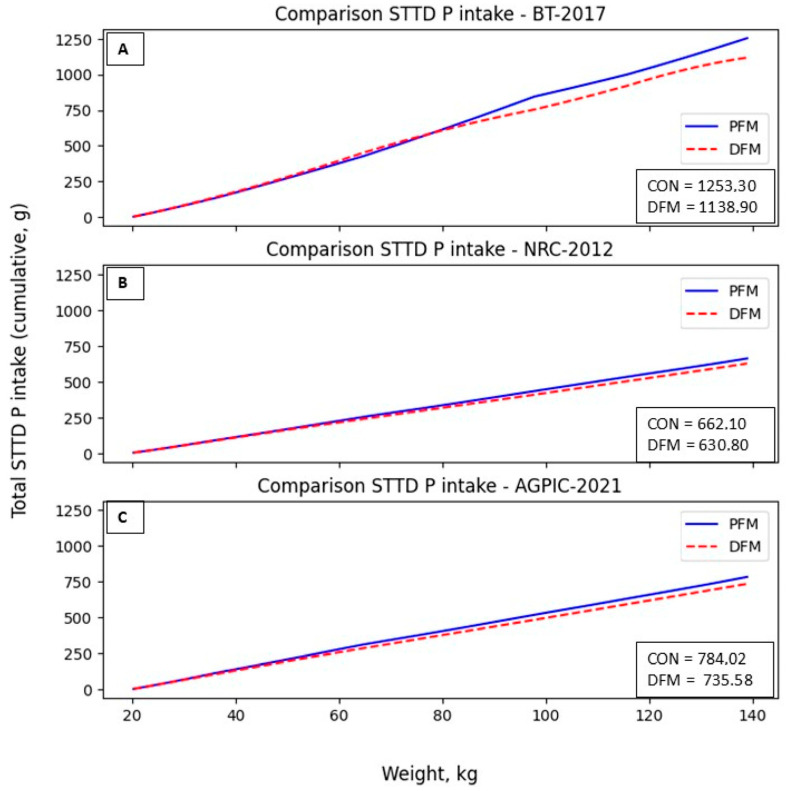
Total STTD P intake in the diet (g) in the proposed scenarios. (**A**): BT-2017; (**B**): NRC-2012; (**C**): AGPIC-2021. Abbreviations: CON = conventional phase feeding model; DFM = daily fit model.

The DFM also reduced phosphorus intake in the simulation. In the BT, NRC, and AGPIC scenarios, the diet’s digestible phosphorus content was reduced by 10.87%, 5.28%, and 6.18%, respectively (Figure 7). Notably, in the NRC and AGPIC scenarios, the differences become more prominent from 70.0 kg of BW, while in the BT scenario, these disparities become evident from 100.0 kg of BW onwards.

### 3.4. Cost Reduction

Table 3 provides a comprehensive overview of the daily fit model’s (DFM) cost-saving potential compared to conventional models across the three proposed scenarios. Notably, the NRC-2012 scenario yields the most substantial cost reduction with a $2.58 decrease in feed costs. This was followed by the AGPIC-2021 and BT-2017 scenarios, which cost $2.27 and $2.04, respectively. The simulations underscore the considerable cost-saving potential of the DFM in optimizing feed expenditure.

## 4. Discussion

### 4.1. Nutrient Reduction

The evident cost reduction among the selected scenarios can be primarily attributed to the reduced excess nutrients in the diet. Conventional phase-fed pigs often receive more nutrients than their requirements during the growing-finishing phase. Typically, these requirements are formulated based on average pig values [18], overlooking individual variations within the phase. Such variations are influenced by age, sex, and genetic potential [21,22]. Strategies for precision feeding have emerged as a promising approach to mitigate this issue by tailoring diets to align more closely with individual animal requirements [9,16,23].

Conventional phase feeding systems typically involve formulating three to five diets, and while increasing feeding phases can help reduce nutrient excess, it also complicates feed management [24]. On the other hand, implementing and managing precision feeding systems are associated with costs and structural modifications. Moreover, utilizing automated feeding systems may only be economically viable for some pig farmers with site-specific economic profitability [25,26]. Nevertheless, these nutrient adjustments have the potential to increase nutrient efficiency [16,23], reduce lysine intake [15], and ultimately lower overall costs [6].

The reduction in CP intake, as depicted in Figure 2 and Figure 3, exemplifies the efficacy of the daily fit model (DFM). The model anticipates the subsequent diet and blends it with the current one, resulting in a reduction in nutrients and, consequently, a cost reduction in feeding. Notably, the most relevant CP decreases are observed in phases 3 to 5, where pigs exhibit higher feed intake. Failure to balance these diets during this period can lead to environmental concerns. In addition to applying the proposed DFM, low-CP diets are worth considering, particularly in the finishing phase. Studies have indicated that low CP diets supplemented with appropriate amino acids do not compromise pig growth performance, nutrient digestibility, or meat quality [27].

While the amino acid requirements of pigs naturally decrease during the growth phase, the diet’s concentration needs to be adjusted [18]. Nevertheless, excess amino acids persist in conventional feeding systems. Notably, the NRC and BT models can estimate SID Lys to maximize average daily gain (ADG) but cannot account for within-herd variation [20]. In the scenarios presented in this study, the maximum lysine reduction reached 7.55% for group-fed pigs. For individual precision-fed pigs, SID lysine reductions can reach up to 26% [15]. Nevertheless, these results still indicate the efficacy of the DFM in improving nutrient efficiency without compromising pig performance.

Excess lysine in the diet, exceeding 4%, has decreased weight gain by 16% and feed efficiency by 5%, with up to 26% of lysine excreted in urine [28]. This excess needs to be more environmentally sustainable and economically viable. As observed with the DFM, reducing the excess of essential amino acids can also lower feeding costs. Conventional models for estimating AA requirements in growing-finishing pigs (NRC-2012 and BT-2017) tend to overestimate lysine requirements compared to the average pig [20].

In summary, the oversupply of nutrients beyond the pig’s requirements can lead to growth depression, reduced feed intake [28], decreased ADFI and ADG [29], and reduced nutrient retention [30]. Once imbalances in AA can further impair growth and feed intake, to improve the efficiency of nutrient utilization in pigs, it is crucial to align nutrient supply as closely as possible with individual animal requirements, thus limiting oversupply [31].

The oversupply of amino acids and phosphorus (P) in pig diets raises environmental concerns. Higher concentrations of urea in the blood plasma [32], limited protein synthesis, increased deamination, increased feed costs, and nitrogen excretion of pigs result from the above-mentioned excesses. Lowering CP diets can effectively reduce urea concentration in blood plasma [33], limit protein synthesis, increase deamination [34], and reduce costs and nitrogen excretion by 1.5% for every percentage unit of CP reduction [15,35], maintaining the growth performance of pigs [36].

### 4.2. Reduction in Total N and STTD P

Reducing total nitrogen (N) in the diet consequently reduces nitrogen excretion. The highest reduction observed in this study was 6.77% in the BT scenario. Other studies have reported even higher reductions in nitrogen intake, ranging from 17% [37] to 25% [6,37] in daily tailored diets compared to conventional phase feeding without compromising pig performance. This reduction is possible because pig diets are traditionally formulated with generous safety margins to ensure maximum population responses [38].

This reduction in nitrogen excretion aligns with findings from other studies. Pomar [16] observed that pigs subjected to group precision feeding excreted 12% less nitrogen than pigs in the three-phase program. Andretta [15] found that nitrogen excretion could be reduced by over 30% when comparing individual precision-feeding pigs to phase-feeding pigs. In essence, precision feeding can enhance nitrogen efficiency [39] and effectively minimize nutrient losses, given that nearly all animals tend to receive more nutrients than they require [13].

Another nutrient frequently present in excess in pig diets is phosphorus. As the third most expensive nutrient in pig diets [40,41], often sourced from non-renewable resources, reducing the digestible P content in diets can have significant implications. In this study, reductions in digestible P intake ranged from 5.28% to 10.87% in the BT, NRC, and AGPIC scenarios, respectively. These reductions align well with results obtained by Zhang [37] and Pomar [16], who reported reductions of 9.7% and 4.4% in P intake, respectively.

Reducing P excretion is also achieved with decreases of 6.6% [16] and 30% [34] reported in other studies. It is crucial to note that P is not fully absorbed from pig diets, with approximately 45% of ingested P being absorbed, 30% retained, and the remaining 15% excreted in urine in diets based on soybean bran and cereals [42]. Phosphorus from swine manure can pose environmental pollution risks and contribute to waterway eutrophication [43]. Hence, precise P estimation and feeding strategies can improve P utilization and enhance the sustainability of swine farming.

Nitrogen, a component of amino acid molecules, and P, considered critical nutrients, have higher environmental pollutant potential [44]. The application of nutritional technologies in pig diets has been shown to reduce pig manure production and N and P nutrient levels compared to control diets [45]. Individual daily feeding programs can decrease nitrogen excretion by 1.5% for each percentage unit of protein intake reduction [15], reducing both N and P excretion [46]. Overall, reducing nutrient oversupply can help mitigate the environmental footprint of pig production in Brazil, resulting in reduced acidification, eutrophication, land occupation, and lower costs [15,35,47,48,49].

### 4.3. Cost Reduction

The proposed DFM demonstrates the potential to reduce feeding costs by nearly 2.4% in the simulations (Table 3). This reduction, although modest, is essential for advancing production especially given the high international demand for pork [50]. This reduction can be attributed to the dilution of the supplied feed. At the beginning of the growth phase, pigs have higher nutrient demands, which gradually decrease as they approach the finishing phase. This cost reduction is mainly influenced by lower protein and phosphorus content, which are the second and third most expensive nutrients.

Feeds are conventionally formulated with ample safety margins and excess nutrients to maximize population responses. Adjusting diets closer to actual requirements will likely lead to reduced excess nutrient intake and, consequently, lower feeding costs [33]. Feeding programs involving individually tailored diets and multi-phase feeding have been shown to result in a 10% reduction in feeding costs compared to conventional feeding programs [15,16,51,52].

Furthermore, along with reduced feed costs, implementing adjusted nutritional levels and novel formulation methods aimed at improving nutrient utilization efficiency and reducing nutrient excretion by pigs is highly recommended due to their cost-effectiveness and applicability [52].

This model can be applied to a user-friendly spreadsheet, which makes it accessible to a broader range of farmers, even to smaller farms that might not have the resources to invest in other advanced precision feeding technologies.

Although the DFM simplifies the precision feeding process, it only considers some complexities of the individual animal’s nutritional requirements. It is applied to any group-phase fed pigs, however, without the use of ractopamine. To improve the model’s robustness, validation in a broad spectrum of scenarios with real-field conditions is recommended. This group-based approach also promises to integrate the model with automatic feeders that provide different diets by pen. Evaluating long-term economic and environmental impacts in different scenarios has potential effects on farm profitability.

## 5. Conclusions

In summary, the proposed DFM demonstrates its potential not only in cost reduction but also in reducing nutrient intake among pigs during the crucial growing-finishing phase. This model’s ability to reduce costs provides a strong economic incentive for pig farmers to adopt precision feeding techniques, leading to cost savings in the industry. Moreover, the reduction in nutrient intake offers promising environmental benefits. Minimizing the excess excretion of nutrients can mitigate the impact of swine manure on the environment on a small to large scale. While more developed models may be available, this simplified approach to anticipating subsequent diets can be applied through a user-friendly spreadsheet. This model is an essential step toward integrating advanced precision feeding technologies that align with the principles of productivity and sustainability of global pig farming. Therefore, further studies might validate this model by comparing different practical conditions and feeder technology.

## Figures and Tables

**Table 1:** Specifications of the simulations.

Phase	Duration of the Phase (days)	Weight Range (kg)
1	24	20–35
2	29	35–60
3	29	60–90
4	16	90–110
5	22	>110

**Table 2:** Models’ description.

Equation	Description
D	Phase duration in days
F	Feed price
I	Feed intake
P	Phase
DFI	Daily feed intake
FP1 e FP2	The price of feed 1 and 2 used
AFI 1 = 100 − PD	Amount of feed 1
PD = (100/d) × (D − 1)	Phase duration
d	Phase day
D	Production day
AFI2 = 100 − AF1	Amount of feed 2

**Table 3:** Comparison of feed costs in the proposed scenarios.

Item ^1^	BT-2017	NRC-2012	AGPIC-2021
Feed cost, $/pig—CON	94.12	108.11	100.80
Feed cost, $/pig—DFM	92.09	105.54	98.53
Feed cost, $/pig (reduction in %)—RED	2.04 (2.17%)	2.58 (2.39%)	2.27 (2.25%)

^1^ Abbreviations: CON = conventional phase feeding model; DFM = daily fit model; RED = reduction.

## Data Availability

The tables contain complementary data, and further information should be addressed to the corresponding author.

## Supplementary Materials

The following supporting information can be downloaded at https://www.mdpi.com/article/10.3390/ani14202922/s1, Table S1: Nutrient requirements for barrows used in the simulations. Table S2: Formulated diets used in the simulations..
