# Supplementary material for: A Simplified Daily Fit Model to Reduce Costs and Nutrient Intake in Growing-Finishing Pigs
Source: Animals (Basel). 2024 Oct 11;14(20):2922. doi: 10.3390/ani14202922 (PMC11503865; doi:10.3390/ani14202922)
Supplement: Supplementary file 1 [file animals-14-02922-s001.zip › animals-3148422-supplementary.pdf]

Supplementary Table S1

Nutrient requirements for barrows used in the simulations.

Scenario	Brazilian Tables[1]						NRC[2]						AGPIC[3]					
Feed	1	2	3	4	5	6	1	2	3	4	5	6	1	2	3	4	5	6
Nutrients																		
ME	3350.000	3350.000	3350.000	3350.000	3350.000	3350.000	3400.000	3400.000	3400.000	3400.000	3400.000	3400.000	3400.000	3400.000	3400.000	3400.000	3400.000	3400.000
STTD P	0.421	0.346	0.279	0.241	0.215	0.183	0.330	0.300	0.260	0.240	0.210	0.200	0.420	0.360	0.300	0.270	0.250	0.220
SID Lysine	2.134	1.326	0.980	0.716	0.704	0.582	1.070	0.960	0.810	0.700	0.590	0.530	1.220	1.030	0.830	0.730	0.670	0.582
SID Methionine	0.363	0.321	0.278	0.242	0.209	0.196	0.310	0.280	0.230	0.200	0.170	0.150	-	-	-	-	-	-
SID Methionine + Cysteine	0.713	0.631	0.547	0.483	0.418	0.391	0.600	0.540	0.460	0.400	0.350	0.320	0.708	0.597	0.481	0.423	0.389	0.337
SID Threonine	0.813	0.695	0.603	0.523	0.453	0.419	0.600	0.580	0.500	0.450	0.390	0.360	0.793	0.670	0.540	0.482	0.442	0.384
SID Thyptophan	0.238	0.214	0.185	0.161	0.139	0.131	0.180	0.160	0.140	0.120	0.110	0.100	0.232	0.185	0.149	0.131	0.121	0.105
SID Valine	0.863	0.738	0.640	0.555	0.481	0.443	0.690	0.620	0.530	0.460	0.400	0.360	0.708	0.700	0.556	0.496	0.456	0.396
SID Isoleucine	0.688	0.588	0.510	0.443	0.383	0.353	0.560	0.500	0.430	0.370	0.320	0.290	0.683	0.577	0.465	0.409	0.375	0.326
SID Leucine	1.251	1.069	0.927	0.805	0.697	0.641	1.070	0.960	0.820	0.710	0.600	0.540	1.232	1.040	0.838	0.745	0.683	0.593
SID Histidine	0.413	0.353	0.306	0.266	0.230	0.210	0.370	0.330	0.280	0.240	0.200	0.180	0.403	0.350	0.282	0.248	0.228	0.198
SID Phenylalanine	0.626	0.535	0.464	0.403	0.349	0.323	0.640	0.570	0.490	0.420	0.360	0.330	-	-	-	-	-	-
SID Phenylalanine + Tyrosine	1.251	1.069	0.927	0.805	0.697	0.641	1.000	0.900	0.760	0.660	0.570	0.510	1.135	0.968	0.789	0.701	0.643	0.558

Abbreviations: ME = Metabolized Energy; SID = Standardized ileal digestibility.

Units: ME= kcal/kg; Other nutrients= %

[1]All requirements were obtained from (Rostagno, 2017)).

[2] All requirements were obtained from NRC (2012).

[3] All requirements were obtained from PIC (2021).

Supplementary Table S2

Formulated diets used in the simulations.

Diets																		
Scenario	Brazilian Tables						NRC						AGPIC					
Feed	1	2	3	4	5	6	1	2	3	4	5	6	1	2	3	4	5	6
Price of Feed*																		
	$ 0.65	$ 0.64	$ 0.46	$ 0.45	$ 0.42	$ 0.40	$ 0.47	$ 0.46	$ 0.43	$ 0.41	$ 0.39	$ 0.38	$ 0.45	$ 0.42	$ 0.40	$ 0.38	$ 0.37	$ 0.35
Nutrients																		
ME	3297.305	3251.765	3241.603	3246.508	3209.390	3163.792	3400.000	3400.000	3400.000	3400.000	3400.000	3400.000	3400.000	3400.000	3400.000	3400.000	3400.000	3400.000
CP	19.120	19.539	15.960	13.964	11.676	8.938	17.883	16.486	14.998	13.596	12.539	11.650	18.375	16.919	16.393	13.932	11.308	10.168
Total Calcium	0.904	0.907	0.524	0.454	0.406	0.335	0.700	0.650	0.570	0.510	0.450	0.420	0.831	0.423	0.233	0.247	0.581	0.570
STTD P	0.403	0.405	0.254	0.254	0.197	0.162	0.330	0.300	0.260	0.240	0.210	0.200	0.420	0.360	0.300	0.270	0.250	0.220
SID Lysine	1.423	1.446	0.835	0.726	0.630	0.571	1.070	0.960	0.810	0.700	0.590	0.530	1.220	1.030	0.830	0.730	0.670	0.582
SID Methionine	0.442	0.448	0.251	0.218	0.189	0.198	0.343	0.296	0.230	0.212	0.200	0.190	0.440	0.346	0.232	0.205	0.203	0.168
SID Methionine + Cysteine	0.701	0.711	0.493	0.436	0.378	0.352	0.600	0.540	0.462	0.430	0.410	0.391	0.708	0.597	0.481	0.424	0.389	0.341
SID Threonine	0.891	0.905	0.543	0.472	0.410	0.263	0.600	0.580	0.500	0.450	0.390	0.360	1.339	0.787	0.540	0.482	0.817	0.770
SID Thyptophan	0.297	0.302	0.167	0.145	0.126	0.118	0.180	0.160	0.140	0.120	0.110	0.100	0.232	0.185	0.168	0.135	0.121	0.105
SID Valine	0.762	0.780	0.663	0.575	0.473	0.347	0.702	0.647	0.592	0.537	0.497	0.461	0.761	0.700	0.687	0.577	0.460	0.412
SID Isoleucine	0.695	0.713	0.584	0.495	0.391	0.264	0.648	0.587	0.527	0.466	0.422	0.383	0.683	0.621	0.607	0.494	0.375	0.326
SID Leucine	1.395	1.424	1.317	1.195	1.045	0.863	1.442	1.364	1.289	1.211	1.156	1.106	1.440	1.361	1.346	1.194	1.029	0.963
SID Histidine	0.438	0.448	0.393	0.346	0.290	0.222	0.434	0.403	0.372	0.341	0.319	0.299	0.443	0.412	0.406	0.347	0.282	0.257
SID Phenylalanine	0.807	0.826	0.702	0.607	0.496	0.361	0.773	0.710	0.647	0.584	0.538	0.497	0.804	0.742	0.729	0.609	0.479	0.426
SID Phenylalanine + Tyrosine	1.408	1.442	1.231	1.068	0.876	0.642	1.287	1.183	1.081	0.978	0.903	0.836	1.391	1.285	1.264	1.061	0.841	0.752
Total Nitrogen	2.655	2.716	2.232	1.939	1.605	1.204	2.861	2.638	2.400	2.175	2.006	1.864	2.739	2.463	2.293	1.934	1.570	1.384

Abbreviations: ME = Metabolized Energy; CP = Crude Protein; STTD P = Standardized Total Tract Digestible Phosphorus; SID = Standardized Ileal Digestibility.

Units: ME= kcal/kg; Other nutrients= %. * The conversion from Brazilian reais to US dollars was performed using an exchange rate of 5.05 reais per dollar.
